# S-Equol Ameliorates Menopausal Osteoarthritis in Rats through Reducing Oxidative Stress and Cartilage Degradation

**DOI:** 10.3390/nu16142364

**Published:** 2024-07-21

**Authors:** Yu-Chen Hu, Tzu-Ching Huang, Li-Wen Huang, Hsiao-Ling Cheng, Bau-Shan Hsieh, Kee-Lung Chang

**Affiliations:** 1 Department of Biochemistry, School of Medicine, College of Medicine, Kaohsiung Medical University, Kaohsiung 807378, Taiwan; huyujena@gmail.com (Y.-C.H.); huangtavia@gmail.com (T.-C.H.); hsiehbs@gmail.com (B.-S.H.); 2 Graduate Institute of Medicine, College of Medicine, Kaohsiung Medical University, Kaohsiung 807378, Taiwan; 3 Department of Medical Laboratory Science and Biotechnology, College of Health Sciences, Kaohsiung Medical University, Kaohsiung 807378, Taiwan; lewehu@cc.kmu.edu.tw; 4 Department of Pharmacy, Kaohsiung Municipal Min-Sheng Hospital, Kaohsiung 802511, Taiwan; chenghl.tanya@gmail.com

**Keywords:** S-equol, isoflavones, osteoarthritis, degenerative joint disease, menopause

## Abstract

Osteoarthritis (OA) is a chronic degenerative disease leading to articular cartilage destruction. Menopausal and postmenopausal women are susceptible to both OA and osteoporosis. S-equol, a soy isoflavone-derived molecule, is known to reduce osteoporosis in estrogen-deficient mice, but its role in OA remains unknown. This study aimed to explore the effect of S-equol on different degrees of menopausal OA in female Sprague–Dawley (SD) rats induced by estrogen deficiency caused by bilateral ovariectomy (OVX) combined with intra-articular injection of mono-iodoacetate (MIA). Knee joint histopathological change; serum biomarkers of bone turnover, including N-terminal propeptide of type I procollagen (PINP), C-terminal telopeptide of type I collagen (CTX-I) and N-terminal telopeptide of type I collagen (NTX-I); the cartilage degradation biomarkers hyaluronic acid (HA) and N-terminal propeptide of type II procollagen (PIINP); and the matrix-degrading enzymes matrix metalloproteinases (MMP)-1, MMP-3 and MMP-13, as well as the oxidative stress-inducing molecules nitric oxide (NO) and hydrogen peroxide (H_2_O_2_), were assessed for evaluation of OA progression after S-equol supplementation for 8 weeks. The results showed that OVX without or with MIA injection induced various severity levels of menopausal OA by increasing pathological damage, oxidative stress, and cartilage matrix degradation to various degrees. Moreover, S-equol supplementation could significantly reduce these increased biomarkers in different severity levels of OA. This indicates that S-equol can lessen menopausal OA progression by reducing oxidative stress and the matrix-degrading enzymes involved in cartilage degradation.

## 1. Introduction

Osteoarthritis (OA) is a chronic degenerative and disabling disease characterized by the wear and tear of articular cartilage and inflammation of the joints. It affected approximately 654.1 million people worldwide in 2020 [1]. Aging is one of the most prominent risk factors for OA, and the prevalence in women of >50 years of age is higher than in men at around 22% and 12%, respectively [1]. OA strikes women more often after menopause, and many studies have indicated that menopause is associated with OA’s onset and progression owing to the deficiency of estrogen [2,3,4]. Osteoporosis is characterized by reduced bone mineral density (BMD) and is often found in menopausal women. Studies have shown osteoporosis is causally linked to the risk of OA as patients with OA had lower BMD [5], and most menopausal and postmenopausal women with low BMD are more susceptible to OA lesions [6]. In addition, evidence suggests the medications used to treat osteoporosis can reduce OA progression and disease symptoms [7]. Notably, postmenopausal women also have a 60% increased risk of developing metabolic syndrome [8], which is related to obesity, dyslipidemia, hyperglycemia and hypertension, and contributes to the occurrence of metabolic OA [9,10]. Addressing obesity and dyslipidemia in postmenopausal women can also improve OA condition.

Hormone replacement therapy has been proven to alleviate the symptoms of menopause and post-menopause women. Unfortunately, estradiol therapy can increase the risk of developing breast cancer and ovarian cancer, which outweighs the benefits [11,12]. Therefore, alternative or nutritional intake strategies for overcoming menopausal estrogen deficiency become necessary. Soy isoflavones, soybean compounds, are structurally similar to estrogen and have been proven to decrease menopausal symptoms and hormone-related diseases [13,14]. S-equol [7-hydroxy-3-(4′-hydroxyphenyl)-chroman] (Structure in Supplementary Figure S1) is a metabolite of daidzein, a soy isoflavone, produced by microflora in the intestine. S-equol is stable, easily absorbed, and has lower clearance than its precursor molecule, daidzein [15]. Simultaneously, it shows more vigorous estrogenic activity and potent antioxidant activity than any other isoflavone or isoflavone-derived metabolite [16,17,18,19,20,21]. It is noted that not all healthy adults can produce S-equol when challenged with soy protein, just only 25–30% of the adult population in Western countries [22,23], and lower than 50–60% of the adult population in Japan, Korea, and China [24,25,26]. Therefore, studies have demonstrated that taking dietary S-equol supplements can reduce osteoporosis in mice with estrogen deficiency by promoting osteoblast formation [27,28]. A daily 10 mg S-equol intake can prevent bone density loss in menopausal women [29]. Our previous in vitro study also showed that S-equol can protect chondrocytes from oxidative-induced matrix degradation and apoptosis, which are commonly found in OA [30]. Additionally, studies have reported that equol can suppress rheumatoid arthritis development and rheumatoid arthritis-induced bone erosion in rats [31]. Low equol production could result in a higher risk of finger osteoarthritis in menopausal and postmenopausal women [32]. Accordingly, we hypothesized that S-equol may benefit menopausal OA.

Ovariectomy (OVX) animal models are usually used in experiments for exploring the effects of estrogen deficiency [33]; moreover, OVX can cause OA in several rat models [34,35,36]. The intra-articular injection of mono-iodoacetate (MIA) is also common and a good method to induce OA in rats, leading to articular chondrocyte death, proteoglycan matrix loss, and stiffness which are similar to those found in human OA [37,38]. In this study, we aimed to investigate whether S-equol is effective against menopausal OA in the OVX rat model. Herein, we use the OVX rat model without or accompanied by a low (0.1 mg) or high dose (1 mg) of MIA by intra-articular injection to induce different degrees of OA severity, including mild (OVX), moderate (OVX-M), and severe (OVX-S) status, since reports have shown the effectiveness of OA treatment is dependent on the timing and disease severity [39,40,41,42,43]. For evaluation, the knee joint OA onset and progression parameters including body weight, lipids, histopathological changes, cartilage degradation, matrix-degrading enzymes, bone turnover, and oxidative stress were analyzed after daily oral S-equol supplementation for 8 weeks.

## 2. Materials and Methods

### 2.1. Animals

Seventy-two female Specific Pathogen Free (SPF) Sprague–Dawley (SD) rats, aged six weeks and weighing 210–240 g, were procured from BioLASCO Taiwan Co., Ltd. (Charles River Technology, Taipei, Taiwan) and kept in a controlled environment with a 12 h light–dark cycle, a temperature maintained at 23 ± 1 °C and humidity at 40–60%. All experiments were conducted in adherence to the National Institutes of Health Guide for the Care and Use of Laboratory Animals, the Animal Care and Use Program of the Kaohsiung Medical University Center for Laboratory Animals, and the ARRIVE guidelines for reporting animal research. Two rats were housed in one cage and given food and water ad libitum. The rats were fed a standard rodent chow that contained 0.9% calcium and 0.7% phosphate (Altromin, Lage, Germany). The rats were randomly allocated to a treatment group, as explained below. The Institutional Animal Care and Use Committee (IACUC) of Kaohsiung Medical University reviewed and approved the animal use protocol with approval number 107214 on 7 May 2019.

### 2.2. Experimental Design

Upon arrival at the laboratory, the female SD rats were placed into standard cages, with two rats per cage in accordance with the instructions of the Animal Care and Use Program of Kaohsiung Medical University. When the estimated weight of the experimental rats exceeded 400 g at the end of the experiment, having only two rats per cage avoids overcrowding in their living space. After a 7 day adaptation period, the rats were randomly divided into 8 groups, with 9 rats in each group: (1) sham group, (2) sham + S-equol group, (3) ovariectomy (OVX) group, (4) OVX + S-equol group, (5) OVX-M (low dose, 0.1 mg MIA) group, (6) OVX-M + S-equol group, (7) OVX-S (high dose, 1 mg MIA) group, and (8) OVX-S + S-equol group. To induce OA, the rats were anesthetized with isoflurane (Panion & BF Biotech Inc., Taoyuan, Taiwan), and the concentration was gradually increased from 0.5% to 4% every 5 min. Eighteen rats underwent sham ovariectomy (sham), and fifty-four rats underwent bilateral ovariectomy (OVX) to induce estrogen depletion as an experimental animal model of estrogen depletion-induced OA [33]. Mild (OVX), moderate (OVX-M), and severe (OVX-S) arthritis were then induced. A total of 18 mild (OVX) rats were injected intra-articularly with 20 μL of sterile saline (0.9% NaCl), and 18 moderate (OVX-M) or 18 severe (OVX-S) rats were injected intra-articularly with 20 μL of 0.1 or 1 mg MIA (Sigma-Aldrich, St. Louis, MO, USA) dissolved in sterile saline. After the surgery, the sham group, OVX group, OVX-M group, and OVX-S group were divided into two subgroups, respectively, without or with oral supplementation of 2 mg/kg/d S-equol (Cayman Chemical Company, Ann Arbor, MI, USA) for eight weeks. At the end of the experiment, the rats were euthanized with CO_2_, and blood and knee joints were collected for further analysis.

### 2.3. Body Weight

The animals’ body weight was measured weekly to track weight change and monitor their health condition. The total weight gain was calculated by subtracting the initial body weight at the start of the treatment from the final body weight before euthanasia.

### 2.4. Histopathologic Analysis of Cartilage

The cut joints were embedded in an optimal cutting temperature (O.C.T) embedding compound and kept at −80 °C until further examination. The joint was sliced sagittally into 5 μm thick sections by cryostat (Leica CM1950, Leica Biosystems, Nussloch, Germany) and stained with Safranin O/Fast green (Sigma-Aldrich, St. Louis, MO, USA), which was analyzed as previously described [44]. The degree of knee cartilage pathological damage in each section was evaluated using the International Osteoarthritis Research Society International (OARSI) scoring system [45]. In this system, cartilage matrix loss in OA is graded from 0 to 6, and cartilage damage is classified from 0 to 4. The scores are multiplied, resulting in a score from 0 to 24, with a higher score indicating more severe cartilage tissue damage [46]. All sections were performed in different staining batches, and each batch was examined by two experienced observers who were blinded to the study. After multiplying, the scores were averaged to yield a cartilage pathology score. To account for differences in scoring among observers due to varying levels of expertise, interpretation, or subjective judgment, introducing observers as a random effect helps manage the variability introduced by different individuals assessing the slide samples.

### 2.5. Serum Sample Collection

At the end of the study, the rats were euthanized and blood samples were collected followed by centrifugation at 3000× *g* for 15 min to obtain the serum. The serum samples were divided into aliquots and stored at −80 °C until further analysis.

### 2.6. Enzyme-Linked Immunosorbent Assay

Rat sandwich enzyme-linked immunosorbent assay (ELISA) kits were used to detect the levels of cartilage degradation biomarkers hyaluronic acid (HA) (cat. no. E0034Ge; Bioassay Technology Laboratory, Jiaxing, Zhejiang, China) and N-terminal propeptide of type II procollagen (PIINP) (cat. no. MBS2024444) (MyBioSource, San Diego, CA, USA); bone turnover biomarkers N-terminal propeptide of type I procollagen (PINP) (cat. no. MBS4501851), C-terminal telopeptide of type I collagen (CTX-I) (cat. no. MBS458687), and N-terminal telopeptide of type I collagen (NTX-I) (cat. no. MBS165137) (MyBioSource, San Diego, CA, USA); and matrix-degrading enzymes matrix metalloproteinase (MMP)-1 (cat. no. MBS2701169), MMP-3 (cat. no. MBS2701197), and MMP-13 (cat. no. MBS2701183) (MyBioSource, San Diego, CA, USA) in the serum of each group (n = 9 per group). The ELISA procedure was strictly performed according to the manufacturer’s instructions. All samples were examined in triplicate within each assay.

### 2.7. Serum Cholesterol and Triglyceride Analysis

The serum levels of cholesterol were measured by the enzymatic endpoint method using a cholesterol kit (cat. no. CH7945 Randox Laboratories Ltd., County Antrim, UK). Triglyceride levels in the serum were also measured by the GPO-PAP method using a triglyceride kit (cat. no. TR210 Randox Laboratories Ltd., County Antrim, UK). The final formed color products of both tests were measured spectrophotometrically. The procedure was strictly performed according to the manufacturer’s instructions. All samples were examined in triplicate within each assay.

### 2.8. Serum H_2_O_2_ and NO Determination

Concentrations of hydrogen peroxide (H_2_O_2_) in the serum were measured by a commercially available hydrogen peroxide assay kit (cat. no. ab102500; Abcam, Cambridge, UK). Serum nitric oxide (NO) bioavailability was measured using a commercially available nitrate/nitrite colorimetric assay kit (cat. no. 780001, Cayman Chemical, Ann Arbor, MI, USA). The measurements were performed according to the manufacturer’s instructions. All samples were examined in triplicate within each assay.

### 2.9. Statistical Analysis

The D’Agostino–Pearson omnibus normality test was performed to determine if the data followed a normal distribution. All variables exhibited normal distribution and were then subjected to one-way ANOVA with Tukey post hoc tests. All statistical analyses were performed using GraphPad Prism 10 (version 10.2.3; Boston, MA, USA). The results are presented as mean ± standard deviation (SD). A *p*-value < 0.05 was considered statistically significant. The actual numerical values of the mean ± S.D. of each group and 95% CI between each group are shown in Supplementary Figures S2 and S3.

## 3. Results

### 3.1. Body Weight and Physiologic Parameters

**Figure 1:**
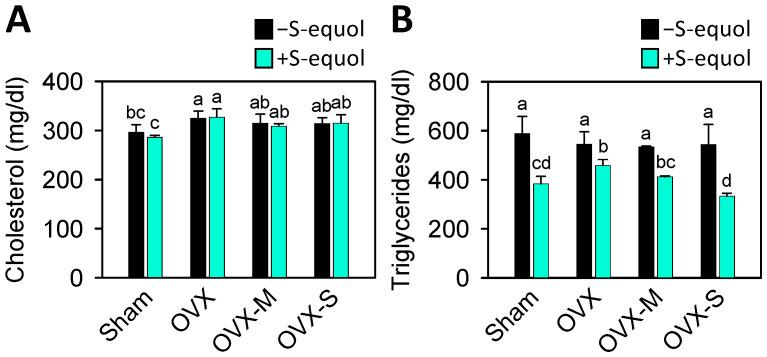
Serum levels of cholesterol and triglycerides in the control sham and OVX-OA rats without or with S-equol supplementation. The details of the OVX-OA rat groups are described in Section 2.2. After S-equol supplementation for 8 weeks, levels of (**A**) cholesterol and (**B**) triglycerides in serum were analyzed by enzymatic kits. The results are presented as means ± S.D. (*n* = 9). Groups with different letters show a significant difference, while those with the same letter show no significant difference. A *p*-value < 0.05 was considered statistically significant.

At the end of an 8-week supplementation, the OVX rats’ body weight significantly increased compared to the sham group. The increase rates of the sham, OVX, OVX-M, and OVX-S groups were 135.9 ± 1.3%, 165.9 ± 3.1%, 178.2 ± 4.2% and 175.5 ± 0.8%, respectively (Table 1). These results were consistent with the concept that estrogen deficiency would increase body weight. S-equol supplementation significantly decreased body weight in the sham and OVX-M groups compared to their respective groups without S-equol supplementation. Rats in the OVX-only group had higher serum cholesterol than the sham group, but not those with OVX and MIA injections (OVX-M and OVX-S groups). S-equol supplementation did not change serum cholesterol levels in any group. The treatment of OVX and MIA injection did not alter serum triglyceride levels, while S-equol supplementation decreased serum triglyceride levels in all groups (Figure 1).

### 3.2. Macroscopic Observation and Knee-Joint Histopathologic Changes

**Figure 2:**
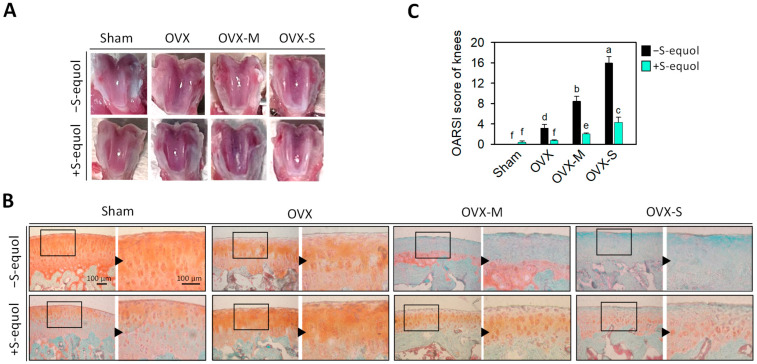
Articular cartilage morphology and histology in the control sham and OVX-OA rats without or with S-equol supplementation. The details of the OVX-OA rat groups are described in Section 2.2. (**A**) Images of the articular surfaces of the femoral groove. (**B**) Joint cartilage histomorphological changes stained with Safranin O/Fast green. Red indicates proteoglycan. Scale bar: 100 μm. (**C**) OARSI score of each joint. The results are presented as means ± S.D. (*n* = 9). Groups with different letters show a significant difference, while those with the same letter show no significant difference. A *p*-value < 0.05 was considered statistically significant.

Figure 2A shows representative images of the macroscopic observations of the femoral condyle of rats after 8 weeks of S-equol supplementation. The cartilage surface in the untreated sham group was smooth and regular in color, and no cartilage defects or osteophytes were observed. In contrast, the groups of OVX, OVX-M, and OVX-S without S-equol supplementation had ulcers on the articular surface, chondral softening, gloss loss, and subchondral bone exposure as well as a partially sunken femoral condyle surface. These issues worsened with the severity of OA in the OVX, OVX-M, or OVX-S groups. In the S-equol supplementation groups, the cartilage surface became smoother, the chondral softening was decreased, and the sunken surface of the femoral condyle was reduced.

In Safranin O/Fast green staining, the Safranin O binds to acidic polysaccharides in cartilage, appearing as a red color, while Fast green binds to tissues lacking polysaccharides, appearing as a green or blue color. Figure 2B presents Safranin O/Fast green staining images of the knee joints of different rat groups along with their Osteoarthritis Research Society International (OARSI) scores (Figure 2C). The femur cartilage surfaces were smooth in the untreated sham group, and the cartilage structure and layers were well-defined. The chondrocytes were arranged regularly, and Safranin O staining was uniformly distributed (red color). The OVX groups without S-equol supplementation had cartilage damage with Safranin O staining (red color) loss, which increased in severity in the OVX, OVX-M, and OVX-S groups so that almost no Safranin O staining was observed in the OVX-M and OVX-S groups; meanwhile, the Fast green staining was positive, suggesting the absence of acidic proteoglycan cartilage. On the other hand, the S-equol supplementation in each group lessened the decrease in Safranin O staining and the increase in Fast green staining. Figure 2C shows the OVX rats had higher OARSI scores than the sham group; moreover, the score increased in degree with the severity of the MIA concentration administered via intra-articular injection, suggesting that the severity of OA status meets the requirements of this study. S-equol supplements in the OVX, OVX-M, and OVX-S groups significantly decreased the OARSI scores compared to their respective groups without supplementation. This indicates that S-equol was effective for mild, moderate, and severe OA of OVX rats. Supplementary Figure S4 displays additional tissue section images of cartilage.

### 3.3. Serum Biomarkers of Bone Turnover

Normal subchondral bone turnover, intact chondrocyte function, and ordinary biomechanical stresses are crucial for maintaining the structural integrity of articular cartilage, as suggested by experimental and clinical observations [47]. Biochemical markers of bone turnover are generally categorized into bone formation and resorption markers. Serum N-terminal propeptide of type I procollagen (PINP) is associated with bone and osteophyte formation [48]. In contrast, serum C-terminal telopeptide of type I collagen (CTX-I) and serum N-terminal telopeptide of type I collagen (NTX-I) are associated with bone resorption [49,50,51]. Herein, serum levels of PINP, CTX-I, and NTX-I were analyzed, since reports have shown that bone status is associated with menopausal OA progression [5,7].

**Figure 3:**
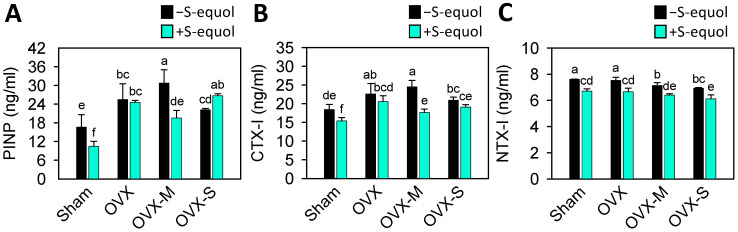
Serum levels of bone turnover biomarkers of PINP, CTX-I, and NTX-I in the control sham and OVX-OA rats without or with S-equol supplementation. The details of the OVX-OA rat groups are described in Section 2.2. After S-equol supplementation for 8 weeks, levels of (**A**) PINP, (**B**) CTX-I, and (**C**) NTX-I in serum were analyzed by enzyme-linked immunosorbent assay. The results are presented as means ± S.D. (*n* = 9). Groups with different letters show a significant difference, while those with the same letter show no significant difference. A *p*-value < 0.05 was considered statistically significant.

The levels of PINP increased in the OVX, OVX-M, and OVX-S groups as compared with the sham group (Figure 3A), indicating the OVX-caused estrogen deficiency might stimulate subchondral osteophyte formation. The PINP levels were increased in the OVX-S group after S-equol supplementation compared with the OVX-S group, but not in the other groups, even decreasing in the sham and OVX-M groups. On the other side, the levels of bone resorption marker CTX-I of all the OVX groups were higher than in the sham group (Figure 3B), while the NTX-I levels of the OVX-M and OVX-S groups were lower than in the sham group (Figure 3C). Supplementation of S-equol decreased CTX-I in the sham and OVX-M groups and decreased NTX-I levels in all the OVX groups, suggesting that S-equol can inhibit bone resorption.

### 3.4. Serum Biomarkers of Cartilage Degradation

**Figure 4:**
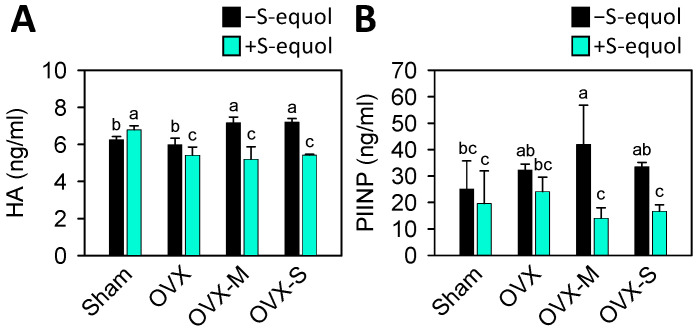
Serum levels of HA and PIINP cartilage degradation biomarkers in the control sham and OVX-OA rats without or with S-equol supplementation. The details of the OVX-OA rat groups are described in Section 2.2. After S-equol supplementation for 8 weeks, levels of (**A**) HA and (**B**) PIINP in serum were analyzed by enzyme-linked immunosorbent assay. The results are presented as means ± S.D. (*n* = 9). Groups with different letters have a significant difference, while those with the same letter show no significant difference. A *p*-value < 0.05 was considered statistically significant.

Serum levels of hyaluronic acid (HA) and N-terminal propeptide of type II procollagen (PIINP) are associated with OA disease severity; the higher the levels are, the more severe the OA [52]. The results showed that OVX-M and OVX-S rats had higher HA levels than the sham rats (Figure 4A), while OVX-M also had higher PIINP levels than the sham rats (Figure 4B). The S-equol supplementation significantly decreased HA levels in the OVX, OVX-M, and OVX-S groups, and PIINP levels in the OVX-M, and OVX-S groups. This suggests S-equol supplementation may diminish OA progression.

### 3.5. Serum Biomarkers of Matrix-Degrading Enzymes

**Figure 5:**
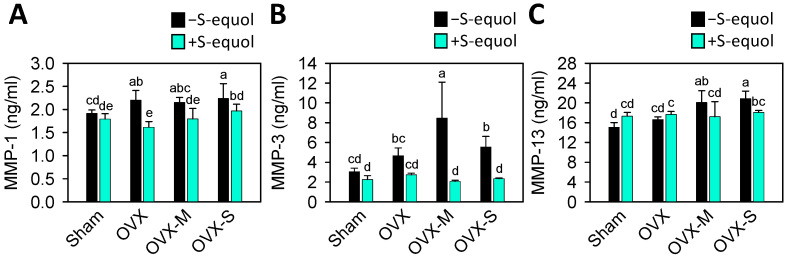
Serum levels of MMP-1, MMP-3, and MMP-13 matrix-degrading enzymes in the control sham and OVX-OA rats without or with S-equol supplementation. The details of the OVX-OA rat groups are described in Section 2.2. After S-equol supplementation for 8 weeks, levels of (**A**) MMP-1, (**B**) MMP-3, and (**C**) MMP-13 in serum were analyzed by enzyme-linked immunosorbent assay. The results are presented as means ± S.D. (*n* = 9). Groups with different letters show a significant difference, while those with the same letter show no significant difference. A *p*-value < 0.05 was considered statistically significant.

As is well known, the matrix metalloproteinases (MMP)-1, MMP-3, and MMP-13 participate in the destruction of articular cartilage in OA. MMP-1 (interstitial collagenase) plays an active role in degrading collagen types I, II, and III, while MMP-3 (stromelysin 1) and MMP-13 (collagenase 3) play roles in the breakdown of major cartilage matrix components, including type II collagen and proteoglycan [53,54]. The levels of serum MMPs were analyzed to investigate whether they are involved in menopausal OA progression. As Figure 5 shows, the levels of MMP-1 increased in the OVX and OVX-S groups, and the levels of MMP-3 and MMP-13 increased in the OVX-M and OVX-S groups compared to the sham group. S-equol supplementation reduced MMP-1 in all OVX groups, while the levels of MMP-3 and MMP-13 decreased only in the OVX-M and OVX-S groups. This indicates that OVX induces matrix degradation by matrix-degrading enzymes, that MIA injection worsens it, and that S-equol supplementation can reduce the matrix degradation

### 3.6. Oxidative Stress in Serum

**Figure 6:**
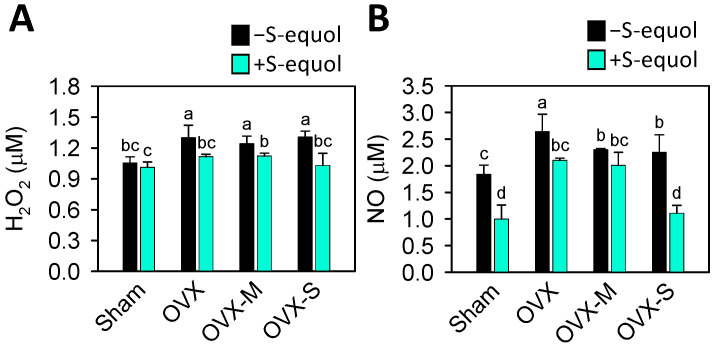
Serum levels of H_2_O_2_ and NO in the control sham and OVX-OA rats without or with S-equol supplementation. The details of the OVX-OA rat groups are described in Section 2.2. After S-equol supplementation for 8 weeks, levels of (**A**) H_2_O_2_ and (**B**) NO were analyzed using a colorimetric assay kit. The results are presented as means ± S.D. (*n* = 9). Groups with different letters show a significant difference, while those with the same letter show no significant difference. A *p*-value < 0.05 was considered statistically significant.

Accumulating studies have demonstrated that increased oxidative stress augments OA progression and is often linked to estrogen deficiency [55]. Nitric oxide (NO), an inflammatory mediator, can drive the development and progression of OA [56]. The serum levels of hydrogen peroxide (H_2_O_2_) and NO were analyzed to explore the role of oxidative stress. Figure 6 indicates increased levels of H_2_O_2_ and NO in all the OVX groups compared to the sham group. After S-equol supplementation, the levels of H_2_O_2_ and NO tended to decrease, showing more effectiveness in the OVX-S group. These results indicate that OVX-caused estrogen deficiency may increase oxidative stress and MIA injection augments it, while S-equol supplementation can reduce it.

## 4. Discussion

The effects of soy isoflavones on menopausal OA are not yet clear. Only several animal studies have reported that soybean isoflavones taken daily reduce OA progression. Studies based on an anterior cruciate ligament transection (ACLT)-caused OA rat model have found that 20 or 40 mg/kg genistein supplementation could increase the collagen and acid glycosaminoglycan content [57,58] and that 30 mg/kg genistein supplementation could prevent articular cartilage damage in an MIA-induced OA rat model [59]. Moreover, reports showed that 20 mg/kg daidzein supplementation had anti-inflammatory and antioxidant effects in an MIA-induced OA rat model [60], and soybean isoflavone limited cartilage degeneration in the OVX rats [61]. As we know, no in vivo studies focus on the effect of S-equol on menopausal OA. Our present study is the first to show that S-equol is effective against menopausal OA in an OVX rat model with MIA injection. The recommended supplement dose of S-equol is 10–30 mg daily for humans to maintain high steady-state S-equol concentrations in the plasma [62]. It is known that the dosage for rats is usually 6.2 times that of humans [63]. With this conversion rate, the experimental rats of this study received a daily 2 mg/kg S-equol supplement to assess its impact on menopausal OA.

After menopause, most women tend to gain weight and become obese, leading to a higher risk of metabolic syndrome [64]. Postmenopausal women with metabolic syndrome often have lower HDL and higher levels of cholesterol and triglycerides [65]. Research has found that individuals with OA are twice as likely to develop dyslipidemia compared to those without OA [66]. Reducing dyslipidemia helps decrease the occurrence of OA. Clinical studies have demonstrated that female equol producers have substantially lower rates of overweight (13.2% vs. 25.7%) and visceral obesity (6.6% vs. 20.7%) compared to non-equol producers [67]. Our study showed that the OVX rats’ body weights significantly increased compared with the sham group, whereas S-equol supplementation might decrease it (Table 1). Moreover, serum triglyceride levels were reduced by S-equol supplementation (Figure 1B). These results indicate that S-equol could reduce obesity and dyslipidemia and consequently improve OA condition.

The development of OA is related to the bone turnover of the subchondral bone and cartilage degradation. In the early stages of OA, the main features include thinning of the subchondral plate, increased porosity, deterioration of subchondral trabeculae, and reduced bone density. In the later stages, subchondral sclerosis and osteophyte formation occur [68]. These changes are associated with cartilage loss and further damage to the joint [50,51]. Enhancement of bone formation with high serum PINP levels is a predictive value for knee OA progression, especially for progressive osteophytosis [48,50]. Our study showed increased levels of PINP in the OVX, OVX-M, and OVX-S groups. S-equol supplementation decreased PINP levels in the sham and OVX-M groups, whereas in the OVX-S group, S-equol supplementation could further augment this increase (Figure 3A). Meanwhile, the bone resorption marker CTX-I levels in the OVX-M and OVX-S groups were increased (Figure 3B). In contrast, the NTX-I levels of the OVX-M and OVX-S groups were lower than in the sham group (Figure 3C). The supplementation of S-equol decreased CTX-I in the sham and OVX-M groups and decreased NTX-I levels in all OVX groups. Considering these inconsistencies in bone turnover biomarkers between groups and with limited bone-related evaluation, it is hard to interpret the presented results since bone turnover is multifactorial and complex. However, our results also showed that bone turnover is crucial to OVX-OA progression, and the effectiveness of S-equol should be further studied to clarify the relation between osteoporosis and OA in menopause.

The extracellular matrix (ECM) of articular cartilage is composed of three types of proteins: collagens (60–86% of dry weight), proteoglycans (15–40% of dry weight), and other non-collagenous proteins [69]. Hyaluronic acid (HA), a non-sulfated glycosaminoglycan (GAG), is a specific serum biomarker of cartilage degeneration and has been implicated in the pathophysiology of knee OA [70]. In addition, in knee and hip OA, HA was found to be associated with an increase in patients’ serum levels and is a predictive value for further radiographic progression [71]. PIINP is one of the two splice forms of type-II procollagen; it is mainly expressed in embryonic cartilage and re-expressed in osteoarthritic cartilage [72]. In their 5-year longitudinal study, Sharif et al. assessed the serum concentration of PIINP in patients with mild-to-moderate knee OA. They found that the average levels of PIINP were higher in patients with progressive disease compared to those with nonprogressive disease [73]. Consistent with these findings, our results indicated that the levels of HA were higher in the OVX-M and OVX-S groups, and the levels of PIINP were elevated in the OVX-M group compared to the sham group. Furthermore, supplementation with S-equol significantly decreased the levels of HA in the OVX-OA groups and PIINP in the OVX-M and OVX-S groups (Figure 4). These results suggest S-equol can protect against cartilage erosion by reducing cartilage degradation.

Cartilage degeneration occurs when MMP-1, MMP-3, and MMP-13 are activated. This activation leads to the degradation of collagen and proteoglycan, disrupting the balance between ECM degradation and synthesis [74]. MMP-1 plays an active role in the degradation of collagen types I, II, and III. In OA, MMP-3 and MMP-13 can break down major cartilage matrix components, including type II collagen and proteoglycan [54,75]. In Figure 5, our results show that in the OVX group, the expression of the MMP-1 matrix-degrading enzyme increased compared to the sham group. Additionally, the combined OVX and MIA groups showed worsened expression of MMP-3 in the OVX-M group and MMP-13 in the OVX-M and OVX-S groups compared to the OVX group. S-equol supplementation reduced the expression of matrix-degrading enzymes in the more severe OVX OA rats. A recent report [76] showed equol had a protective effect against osteoporotic OA in rats, which was associated with the inhibition of the NF-κB signaling pathway. This supports our finding that, as is well known, the NF-κB signaling pathway plays a crucial role in regulating various catabolic enzymes, including MMPs.

It is reported that menopausal OA occurs due to aging and dead chondrocytes [77]. The aging chondrocytes have elevated oxidative stress due to hydroxyl radical (OH^−^), hydrogen peroxide (H_2_O_2_), superoxide anion (O_2_^−^), and nitric oxide (NO), which can promote cell senescence [78,79]. Aging chondrocytes exhibiting low cartilage synthesis activity and high cartilage degradation activity are related to oxidative stress [80,81]. In this study, the results shown in Figure 6 indicate that OVX-caused estrogen deficiency can lead to increased oxidative stress, as evidenced by elevated levels of H_2_O_2_ and NO, which is expected. Additionally, supplementation with S-equol can help reduce oxidative stress and was particularly effective in the more severe OA group.

## 5. Conclusions

The present study demonstrated that in rats, the bilateral OVX with or without an additional injection of MIA into the knee joint resulted in different severity levels of menopausal OA. This was evidenced through macroscopic observation, histopathologic staining of the knee joint, and testing of serum biomarkers for cartilage degradation and oxidative stress. These changes resembled those found in menopausal OA in women. In addition, S-equol supplementation could lessen the menopausal OA progression by reducing the levels of oxidative stress and matrix-degrading enzymes involved in cartilage degradation.

## Figures and Tables

**Table 1:** Changes in body weight in studied groups.

Group (n = 9)	Before Treatment (g)	After Treatment (g)	Increase Rate (%)
Sham	233.7 ± 8.7	317.7 ± 17.2	135.9 ± 1.3 ^e^
Sham + S-equol	240.3 ± 8.7	317.3 ± 13.7	132.0 ± 0.5 ^f^
OVX	231.7 ± 17.3	383.7 ± 15.8	165.9 ± 3.1 ^c^
OVX + S-equol	231.0 ± 10.2	376.3 ± 19.6	162.9 ± 0.7 ^c^
OVX-M	224.3 ± 6.9	400.0 ± 29.1	178.2 ± 4.2 ^ab^
OVX-M + S-equol	243.7 ± 6.8	380.0 ± 12.8	155.9 ± 0.5 ^d^
OVX-S	228.0 ± 5.9	400.3 ± 13.5	175.5 ± 0.8 ^b^
OVX-S+ S-equol	230.0 ± 5.1	405.5 ± 4.5	179.0 ± 1.7 ^a^

The results are presented as means ± S.D. (*n* = 9). Groups with different letters show a significant difference, while those with the same letter show no significant difference. A *p*-value < 0.05 was considered statistically significant. OVX, ovariectomized; OVX-M, OVX rat with 0.1 mg MIA injection; OVX-S, OVX rat with 1 mg MIA injection.

## Data Availability

Data are contained within the article and Supplementary Materials.

## Supplementary Materials

The following supporting information can be downloaded at: https://www.mdpi.com/article/10.3390/nu16142364/s1: Figure S1: Chemical structure of S-equol; Figure S2: The actual numerical values of the mean± S.D. of each group; Figure S3: The actual numerical values of the 95% CI between each group; Figure S4: Joint cartilage histomorphological changes stained with Safranin O/Fast green.
