# Supplementary material for: S-Equol Ameliorates Menopausal Osteoarthritis in Rats through Reducing Oxidative Stress and Cartilage Degradation
Source: Nutrients. 2024 Jul 21;16(14):2364. doi: 10.3390/nu16142364 (PMC11280421; doi:10.3390/nu16142364)

Supplementary Figure S1
Chemical structure of S-equol.

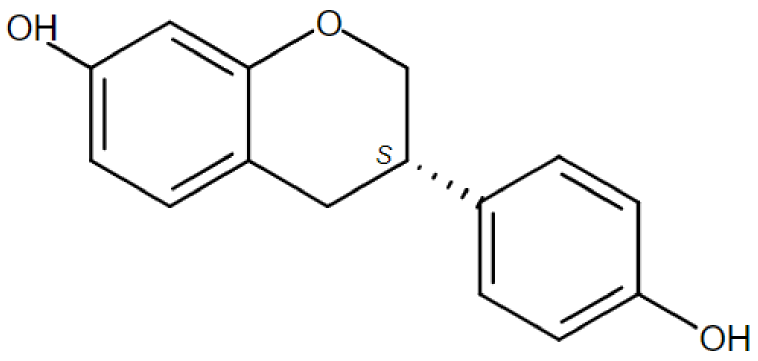

Supplementary Figure S2
The actual numerical values of the mean± S.D. of each group is shown in the table.

	mean± S.D.		
	Cholesterol	Triglycerides	OARSI
Sham	296.3±15.0	588.8±69.4	0.05±0.01
Sham+S-equol	286.1±4.1	383.2±31.1	0.42±0.25
OVX	325.2±14.4	544.4±51.0	3.21±0.69
OVX+S-equol	327.2±16.9	457.9±24.9	0.74±0.14
OVX-M	314.9±18.5	533.9±3.0	8.47±0.97
OVX-M+S-equol	308.7±5.3	411.8±3.7	2.08±0.13
OVX-S	314.2±11.6	543.2±81.8	15.96±1.24
OVX-S+S-equol	314.9±17.5	332.9±12.4	4.33±0.97

	mean± S.D.				
	PINP	CTX-I	NTX-I	HA	PIINP
Sham	16.7±3.9	18.4±1.4	7.61±0.01	6.25±0.17	25.1±10.6
Sham+S-equol	10.5±1.6	15.4±0.9	6.70±0.18	6.79±0.22	19.7±12.3
OVX	25.5±5.1	22.6±2.8	7.52±0.24	5.99±0.34	32.3±2.3
OVX+S-equol	24.5±0.6	20.6±1.6	6.65±0.29	5.42±0.44	24.1±5.5
OVX-M	30.7±4.3	24.5±1.7	7.14±0.22	7.17±0.30	42.0±14.8
OVX-M+S-equol	19.6±2.4	17.7±0.9	6.41±0.10	5.20±0.68	14.0±4.0
OVX-S	22.2±0.5	20.9±0.8	6.94±0.05	7.20±0.19	33.5±1.7
OVX-S+S-equol	26.7±0.6	19.1±0.6	6.12±0.30	5.42±0.04	16.7±2.5

	mean± S.D.				
	MMP-1	MMP-3	MMP-13	H_2O_2	NO
Sham	1.91±0.08	3.03±0.37	15.1±0.9	1.05±0.06	1.84±0.17
Sham+S-equol	1.79±0.12	2.25±0.39	17.3±0.7	1.01±0.05	1.00±0.26
OVX	2.20±0.21	4.67±0.78	16.6±0.6	1.30±0.12	2.64±0.32
OVX+S-equol	1.62±0.12	2.74±0.16	17.7±0.6	1.12±0.02	2.10±0.04
OVX-M	2.15±0.11	8.46±3.65	20.1±2.4	1.24±0.07	2.30±0.02
OVX-M+S-equol	1.80±0.22	2.06±0.12	17.2±3.1	1.12±0.03	2.01±0.25
OVX-S	2.24±0.32	5.54±1.07	20.9±1.5	1.31±0.06	2.25±0.33
OVX-S+S-equol	1.97±0.15	2.34±0.06	18.1±0.4	1.03±0.12	1.11±0.15

Supplementary Figure S3

The actual numerical values of the 95% CI between each group is as shown in the table.

	95% CI		
	Cholesterol	Triglycerides	OARSI
Sham	284.8–307.9	535.5–642.1	0.04–0.06
Sham+S-equol	314.1–336.2	505.2–583.6	2.68–3.74
OVX	300.6–329.1	531.6–536.2	7.72–9.21
OVX+S-equol	305.2–323.1	480.4–606.1	15.01–16.91
OVX-M	282.9–289.2	359.3–407.1	0.23–0.61
OVX-M+S-equol	314.2–340.2	438.8–477.1	0.63–0.85
OVX-S	304.6–312.7	408.9–414.7	1.98–2.18
OVX-S+S-equol	301.4–328.3	323.4–342.5	3.59–5.08

	95% CI				
	PINP	CTX-I	NTX-I	HA	PIINP
Sham	13.6–19.7	17.4–19.5	7.60–7.62	6.12–6.39	17.0–33.3
Sham+S-equol	21.6–29.4	20.4–24.7	7.34–7.70	5.73–6.25	30.5–34.0
OVX	27.4–34.0	23.2–25.8	6.97–7.31	6.94–7.41	30.6–53.3
OVX+S-equol	21.8–22.5	20.3–21.5	6.90–6.97	7.05–7.35	32.2–34.8
OVX-M	9.2–11.7	14.7–16.1	6.56–6.84	6.62–6.96	10.2–29.1
OVX-M+S-equol	24.0–25.0	19.3–21.8	6.43–6.87	5.08–5.75	19.9–28.3
OVX-S	17.7–21.4	17.0–18.4	6.33–6.49	4.67–5.72	10.9–17.1
OVX-S+S-equol	26.3–27.2	18.6–19.6	5.89–6.35	5.39–5.46	14.8–18.6

	95% CI				
	MMP-1	MMP-3	MMP-13	H_2O_2	NO
Sham	1.85–1.97	2.75–3.32	14.4–15.8	1.01–1.10	1.71–1.97
Sham+S-equol	2.04–2.36	4.07–5.27	16.2–17.1	1.21–1.39	2.39–2.89
OVX	2.07–2.23	5.65–11.26	18.2–21.9	1.19–1.30	2.29–2.32
OVX+S-equol	1.99–2.49	4.71–6.37	19.7–22.0	1.26–1.35	2.00–2.51
OVX-M	1.71–1.88	1.95–2.55	16.7–17.9	0.97–1.05	0.79–1.20
OVX-M+S-equol	1.52–1.71	2.62–2.87	17.2–18.1	1.10–1.13	2.07–2.13
OVX-S	1.63–1.97	1.98–2.15	14.8–19.5	1.10–1.14	1.82–2.20
OVX-S+S-equol	1.86–2.08	2.3–2.39	17.8–18.4	0.94–1.12	1.00–1.22

Supplementary Figure S4
Joint cartilage histomorphological changes stained with Safranin O/Fast green. Red indicates proteoglycan.

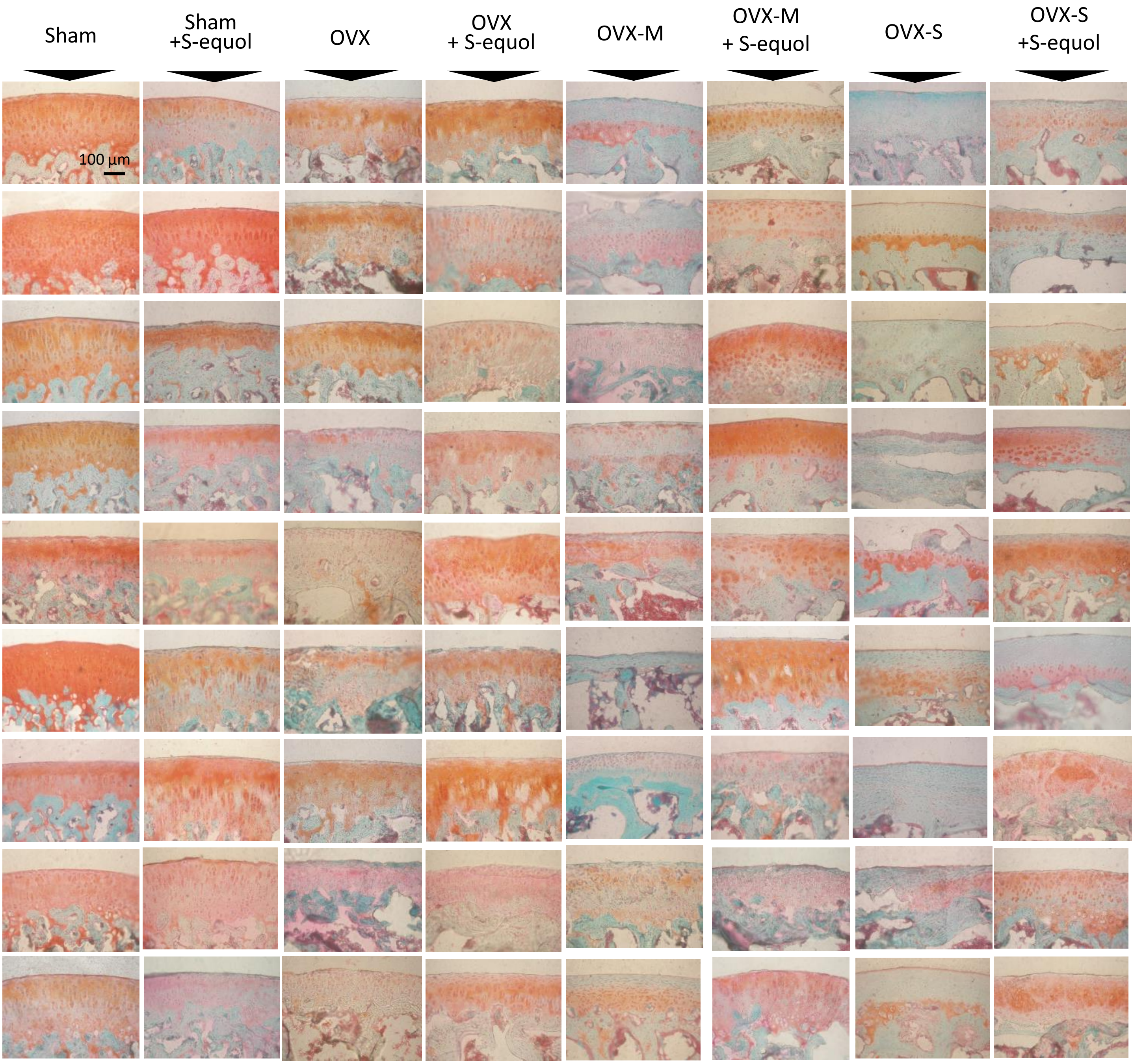

Supplement: Supplementary file 1 [file nutrients-16-02364-s001.zip › nutrients-3109347-supplementary.pdf]
